# Resistance mechanisms and reversal strategies of PD-1/PD-L1 inhibitors in melanoma

**DOI:** 10.3389/fphar.2026.1922343

**Published:** 2026-09-25

**Authors:** Yuhua Li, Jing Wu, Weiming Liao, Chunyan Wang

**Affiliations:** 1 Department of Pharmacy, The First Naval Hospital of Southern Theatre Command, Zhanjiang, Guangdong, China; 2 Department of Pharmacy, No. 971 Hospital of the People’s Liberation Army Navy, Qingdao, Shandong, China; 3 Department of Cardiology, Air Force Hospital of PLA Southern Theater Command, Guangzhou, China

**Keywords:** biomarkers, drug resistance, melanoma, PD-1/PD-L1 inhibitors, resistance-reversal strategies, tumor microenvironment

## Abstract

Programmed cell death protein 1 (PD-1)/programmed death ligand 1 (PD-L1) immune checkpoint inhibitors have redefined the therapeutic landscape of advanced melanoma, establishing a first-line standard of care that markedly prolongs patient survival. Nevertheless, primary and acquired resistance remain the principal barriers to durable clinical benefit: approximately 40%–55% of patients exhibit primary resistance to PD-1 monotherapy inhibitors, while 25%–30% of initial responders develop acquired resistance during treatment. Resistance arises from multidimensional, interconnected mechanisms spanning tumor-intrinsic alterations, immunosuppressive tumor microenvironment remodeling, metabolic and epigenetic reprogramming, and systemic modulators including the gut microbiota. By linking antigen recognition, spatiotemporal regulation, and clinical translation, this review provides a mechanistic framework for understanding PD-1/PD-L1 inhibitor resistance in melanoma. This review aimed to systematically evaluate the evidentiary basis for resistance-reversal strategies—encompassing immune combinations, targeted agents, cellular therapies, and localized interventions—and consolidate hierarchical biomarker systems with translational potential. Critical gaps in clinical applicability, including patient selection, treatment sequencing, and combination-related toxicity, are discussed. Finally, the review highlights unresolved challenges and future directions to guide precision optimization of melanoma immunotherapy.

## 1 Introduction

Melanoma, an aggressive malignancy derived from melanocytic lineage cells, exhibits rising global incidence and disproportionate mortality in advanced stages (Carlino et al., 2021). Landmark advances in immune checkpoint blockade have redefined the therapeutic landscape for advanced disease, establishing programmed cell death protein 1 (PD-1)/programmed death ligand 1 (PD-L1) inhibitors as the first-line standard of care (Ribas and Wolchok, 2018). By disrupting the PD-1/PD-L1 axis, these agents alleviate tumor-mediated immunosuppression and restore endogenous anti-tumor T cell responses, significantly prolonging overall and progression-free survival in treatment-naive patients (Yan et al., 2026). From a pharmacological standpoint, the clinical efficacy ceiling of PD-1/PD-L1 inhibitors is largely attributable to multilayered dysregulation of drug targets and downstream oncogenic signaling networks, necessitating systematic dissection of actionable targets and matched combinatorial regimens (Schoenfeld and Hellmann, 2020).

Clinical benefit with PD-1/PD-L1 inhibitors varies substantially across patient populations, with resistance representing the principal barrier to long-term efficacy. Primary resistance to PD-1 inhibitor monotherapy occurs in 40%–55% of patients, while primary resistance to cytotoxic T-lymphocyte associated protein 4 (CTLA-4) inhibitor monotherapy exceeds 70% (Morad et al., 2021). Even among initial responders, 25%–30% develop acquired resistance during treatment or after discontinuation, culminating in disease progression (Ma et al., 2025). Primary resistance denotes *de novo* lack of clinical response to first-cycle immune checkpoint inhibitor (ICI) administration, whereas acquired resistance describes progression following an initial period of disease control (Chesney et al., 2023). These phenotypes are not mutually exclusive: core mechanisms—including antigen presentation defects and tumor microenvironment (TME) immunosuppression—drive both resistance subtypes, with substantial mechanistic overlap (Ascierto et al., 2023). Collectively, these observations mandate a multidimensional, network-level analysis of resistance mechanisms rather than exclusively focusing on single molecular targets (Ayers et al., 2017).

Resistance operates across four interdependent axes: tumor-intrinsic functional defects, immunosuppressive TME remodeling, metabolic and epigenetic reprogramming, and systemic regulatory factors exemplified by the gut microbiota (Liu et al., 2024). Deep mechanistic dissection is a prerequisite for developing clinically actionable reversal strategies and improving patient outcomes. Preclinical and clinical investigations have identified candidate reversal strategies spanning immune combinations, targeted agents, cellular therapies, and localized interventions, with several regimens demonstrating promising efficacy in early-phase trials. Parallel efforts have established a hierarchical biomarker system for patient stratification, efficacy prediction, and early resistance monitoring, bridging mechanistic insight and clinical decision-making. Subtype-specific resistance profiles—particularly in acral and mucosal melanoma—have emerged as a priority for individualized therapeutic development.

This review systematically delineates the core mechanisms of resistance to PD-1/PD-L1 inhibitors in melanoma, evaluates currently validated or exploratory resistance-reversal strategies, consolidates biomarkers with clinical translational potential, and discusses unsettled challenges and future directions. Through this synthesis, we aim to provide a pharmacological reference for precision optimization of melanoma immunotherapy.

## 2 Mechanisms of resistance to PD-1/PD-L1 inhibitors in melanoma

The occurrence of resistance to PD-1/PD-L1 inhibitors in melanoma arises from the combined actions of multiple factors and dimensions, mainly including five core levels: intrinsic tumor cell mechanisms, TME immunosuppression, metabolic reprogramming, epigenetic reprogramming, and systemic regulatory mechanisms. Mechanisms at all levels are interrelated and interact to jointly promote the formation and maintenance of the resistance phenotype. This chapter elaborates the core resistance mechanisms from five levels, and further analyzes the combinatorial regulatory network across multiple dimensions.

### 2.1 Fundamental resistance mechanisms of melanoma: from primary and acquired drivers to translational patterns

Changes in the intrinsic biological characteristics of tumor cells are one of the core factors mediating resistance to PD-1/PD-L1 inhibitors, mainly involving antigen presentation defects, abnormal activation of signaling pathways, phenotypic transformation and abnormal regulation of molecular expression.

Molecular patterns of resistance to immune checkpoint blockade in melanoma extend beyond genetic alterations. Lauss et al. (2024) demonstrated that non-genetic mechanisms, including epigenetic silencing of antigen processing genes, account for approximately 75% of resistance cases, underscoring a critical limitation of current genomic testing focused solely on somatic mutations. Antigen presentation pathway defect is the key molecular mechanism of primary resistance to PD-1 inhibitors in melanoma, among which β2-microglobulin (B2M) gene mutation and major histocompatibility complex (MHC)-1 molecule loss are the most common (Rodig et al., 2018). As an invariant 12-kDa non-glycosylated light-chain component encoded at chromosome 15q21.1, B2M forms non-covalent complexes with MHC-I heavy-chain molecules. This interaction stabilizes endoplasmic-reticulum-resident peptide-loading assemblies and permits peptide-MHC-I complexes to traffic onto cell surfaces for recognition by CD8^+^ cytotoxic T-cells (Margulies et al., 2023). Notably, some genetic alterations initially arising as acquired-resistance events may become stable intrinsic tumour-cell properties under immunotherapy pressure. Through whole-exome sequencing of melanoma patients with acquired resistance to PD-1 inhibitors, Zaretsky et al. (2016) found that defects in the interferon receptor-related pathway (JAK1/JAK2 mutations) and antigen presentation pathway (B2M mutations) are the main genetic mechanisms mediating acquired resistance. B2M deficiency leads to complete loss of MHC-I on melanoma cells, thus escaping recognition by CD8^+^ T cells, and further mediates irreversible resistance to ICIs (Sade-Feldman et al., 2017). In addition, defects in the antigen processing and presentation pathway of tumor cells also include decreased expression of transporter associated with antigen processing 1/2 (TAP1/2) and abnormal function of HLA-I transcription regulator NLRC5 (Rasmussen et al., 2023).

Abnormal activation of signaling pathways is another important mechanism mediating intrinsic drug resistance. Sustained activation of the MAPK/ERK pathway can directly downregulate tumor antigen expression and upregulate PD-L1 expression, mediating cross-resistance to immunotherapy (Jiang et al., 2013). A transcription factor 4 (TCF4)-dependent gene regulatory network confers resistance to immunotherapy in melanoma. Pozniak et al. (2024) identified TCF4 as a master regulator driving mesenchymal phenotypic transformation, which upregulates immune suppressive ligands and silences antigen processing genes, serving as a central node linking epigenetic reprogramming to phenotypic plasticity. In NRAS-mutant melanoma, abnormal activation of the RAS/RAF/PI3K pathway can directly silence CD40 expression, reduce tumor immunogenicity, and lead to resistance to PD-1 inhibitors (Yan et al., 2021). Loss-of-function mutations in JAK1/2 block interferon-gamma (IFN-γ) signal transduction, leading to acquired resistance to PD-1 inhibitors in melanoma (Zaretsky et al., 2016). Loss of stimulator of interferon genes (STING) pathway function leads to cytoplasmic DNA sensing disorder and decreased type I interferon secretion, which further weakens anti-tumor immune response and mediates resistance to PD-1 inhibitors (Nakamura et al., 2021). In addition, PTEN deletion or inactivating mutation can continuously activate the PI3K/AKT/mTOR pathway, upregulate PD-L1 expression, and mediate resistance to immunotherapy in melanoma (Peng et al., 2016).

Phenotypic transformation and abnormal regulation of molecular expression of tumor cells are also involved in the development of drug resistance. Genomic and transcriptomic analyses of response to anti-PD-1 therapy reveal distinct transcriptional signatures associated with primary and acquired resistance (Hugo et al., 2017). Polycomb repressor complex 2 (PRC2) suppresses interferon-responsive MHC-II expression in melanoma cells and is associated with anti-PD-1 resistance (James et al., 2023). Sustained IFN-γ stimulation may trigger melanoma cellular dedifferentiation, generating the microphthalmia-associated transcription factor^low^/nerve growth factor receptor^high^ (MITF^low^/NGFR^high^) phenotype. This phenotypic shift lowers tumour immunogenicity and contributes to therapeutic resistance against PD-1-targeted inhibitors (Kim et al., 2021). Poly (ADP-ribose) polymerase 14 (PARP14) regulates chromatin remodeling by recruiting histone deacetylases, mediates epigenetic silencing of IFN-γ-related genes, and participates in IFN-γ-driven PD-1 inhibitor resistance (Wong et al., 2023). Abnormal activation of the autophagy pathway in tumor cells can degrade intracellular antigen proteins, reduce MHC-I antigen presentation, and mediate PD-1 inhibitor resistance (Sade-Feldman et al., 2017). Abnormal epigenetic modification can silence immune-related genes through chromatin remodeling and reduce tumor immunogenicity, which is an important regulatory mechanism of phenotypic transformation. Most of the above phenotypic transformations are driven by epigenetic reprogramming, and the specific regulatory mechanisms are detailed in Section 2.4.

A summary of the regulatory mechanisms mediating both intrinsic and acquired resistance to ICIs in melanoma is shown in Table 1. As described above, five core resistance patterns driven by distinct molecular pathways contribute to ICIs failure in melanoma. Furthermore, Figure 1 illustrates the relationship between these phenotypic abnormalities and their core molecular drivers and corresponding therapeutic interventions to provide a roadmap for precision intervention.

**TABLE 1:** Resistance mechanisms to PD-1/PD-L1 inhibitors in melanoma.

Resistance mechanism	Key molecules/genes	Resistance phenotype	Reversal strategy	Evidence level
Antigen presentation defect	B2M mutation/loss; TAP1/2↓; NLRC5↓; HLA-I↓	MHC-I loss → CD8^+^ T cell inability to recognize tumor cells; primary and acquired resistance	B2M screening; combination with TIL/TCR-T; NK cell-based therapy	Established
IFN-γ pathway abnormality				
JAK1/2 loss-of-function	JAK1/JAK2 mutations	Blocks IFN-γ signal transduction; acquired resistance to PD-1 inhibitors	IFN-γ pathway restoration (under investigation)	Preclinical
Oncogenic pathway activation				
MAPK/ERK pathway activation	BRAF/NRAS/RAS mutations; MAP2K2 mutations	Downregulates tumor antigen expression; upregulates PD-L1; mediates cross-resistance	BRAF/MEK inhibitors + PD-1 inhibitors (triplet); MEK/PI3K inhibitors + CD40 agonist	Established (BRAF/MEK inhibitors)
RAS/RAF/PI3K pathway silencing of CD40	NRAS/BRAF mutant; CD40↓	Reduces tumor immunogenicity; PD-1 resistance in NRAS melanoma	RAS/MEK/PI3K inhibitors + CD40 agonist (anti-CD11b+Breg strategy)	Clinical (phase I/II)
PTEN loss/PI3K-AKT-mTOR activation	PTEN deletion/mutation	Upregulates PD-L1; promotes immune resistance	PI3K/AKT/mTOR inhibitors + PD-1 inhibitors (under investigation)	Preclinical
Epigenetic and phenotypic abnormality				
TCF4/MES phenotypic transformation	TCF4-dependent GRN; MES signature	Upregulates immune-suppressive ligands; silences antigen-processing genes	TCF4 pathway inhibitors (exploratory)	Preclinical
Dedifferentiation (IFN-γ-driven)	MITF^low^/NGFR^high^ phenotype	Reduces immunogenicity; mediates PD-1 inhibitor resistance	Prevention of chronic IFN-γ exposure; differentiation-inducing agents	Preclinical
PRC2/EZH2-mediated silencing	EZH2 (PRC2 complex)	Suppresses IFN-responsive MHC-II expression; anti-PD-1 resistance	EZH2 inhibitors + PD-1 inhibitors (exploratory)	Preclinical
PARP14-mediated epigenetic silencing	PARP14 overexpression	Epigenetic silencing of IFN-γ-related genes; acquired resistance	PARP14 inhibitors restore sensitivity to PD-1 blockade	Preclinical
Autophagy and stemness Abnormality				
Autophagy-mediated antigen degradation	Autophagy pathway overactivation	Degrades intracellular antigen proteins; reduces MHC-I presentation	Autophagy inhibitors (e.g., chloroquine; context-dependent)	Preclinical

Evidence levels: Established = phase III/registrational; Clinical = phase I/II, trials; Preclinical = *in vivo*/*in vitro* only. Abbreviations: B2M, β2-microglobulin; TAP, transporter associated with antigen processing; MES, mesenchymal; GRN, gene regulatory network; STING, stimulator of interferon genes; PRC2, polycomb repressor complex 2; EZH2, enhancer of zeste homolog 2; PARP14, poly(ADP-ribose) polymerase 14; MHC, major histocompatibility complex; NK, natural killer.

**FIGURE 1:**
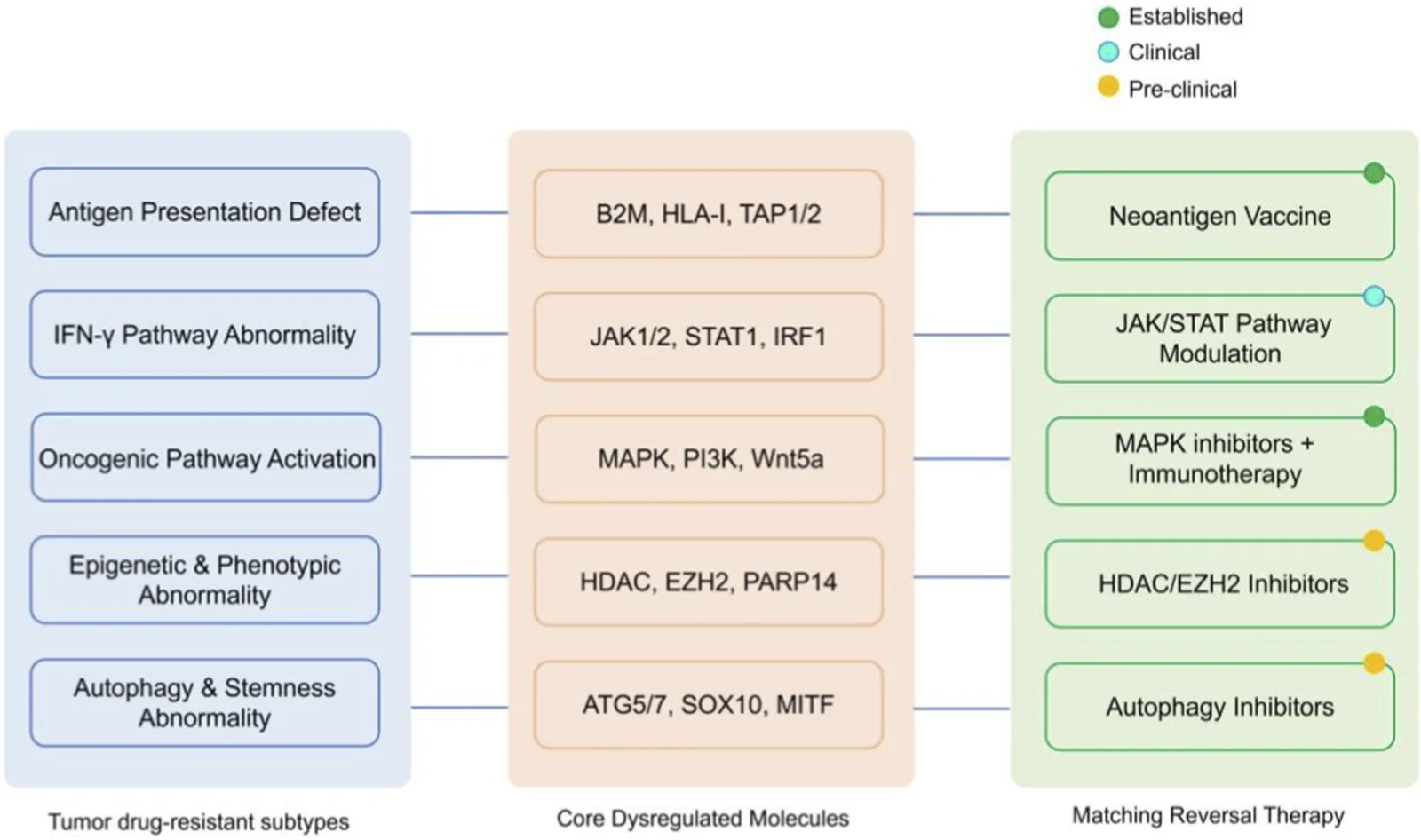
Linear framework linking tumor resistance phenotypes to core dysregulated molecules and matched reversal strategies.

Five predominant resistance subtypes are delineated, each aligned with key molecular drivers and potential therapeutic interventions stratified by translational maturity. Colored circles annotate the evidence level for each regimen: Green denotes established therapies validated by phase III registrational trials; Blue signifies candidates under active phase I/II clinical evaluation; and Orange indicates preclinical approaches.

### 2.2 Immunosuppressive mechanism of the TME

The TME constitutes a complex ecosystem supporting tumour-cell survival and progression. Its immunosuppressive features represent a major extrinsic driver mediating resistance to PD-1/PD-L1 inhibitors. Multiple components, including accumulated immunosuppressive-cell populations, abundant immunosuppressive molecules, acidic metabolic conditions and extracellular-matrix barriers, collectively build up the TME-centred immunosuppressive network that constrains the initiation and persistence of anti-tumour immune responses. This broad immunosuppressive landscape of the melanoma TME has been well documented in previous comprehensive reviews (Özba et al., 2023; Ceci et al., 2020; Routy et al., 2024; Simiczyjew et al., 2020).

Within this suppressive microenvironment, myeloid-derived suppressor cells (MDSCs) serve as key immunosuppressive cellular players in melanoma (Özba et al., 2023; Bitsch et al., 2022). Signals including prostaglandin E_2_ (PGE_2_), granulocyte colony-stimulating factor (G-CSF), granulocyte-macrophage colony-stimulating factor (GM-CSF), and C-C chemokine receptor 5 (CCR5) drive recruitment and functional activation of MDSCs. Once activated, these cells suppress T-cell activity and trigger T-cell apoptotic death via secretion of nitric-oxide synthase, reactive oxygen species (ROS) and arginase-1. MDSCs can activate the STAT3 pathway to promote epithelial-mesenchymal transition, promote angiogenesis and construct a pre-metastatic microenvironment, and their abundance is positively correlated with resistance to ICIs in melanoma (Özba et al., 2023).

After polarization to the M2 phenotype, tumor-associated macrophages (TAMs) secrete a large number of inhibitory cytokines such as interleukin (IL)-10 and TGF-β, directly inhibit CD8^+^ T-cell function, and mediate resistance to PD-1 inhibitors in melanoma (Ceci et al., 2020). Beyond local cellular components, systemic regulators remotely modulate TME immunity: Routy et al. (2024) systematically summarized current understanding of melanoma-microbiota interactions and highlighted their potential to shape TME immune landscapes. Distinct cancer-associated fibroblast (CAF) subsets release immunoinhibitory mediators including CXCL12, CXCL14 and IL-6. These cells also assemble collagen-rich physical barriers and engage TGF-β signalling cascades, which drives resistance to anti-PD-1/anti-PD-L1-based immunotherapy in melanoma (Simiczyjew et al., 2020). Such spatially segregated immunosuppressive microenvironments originate from progressive clonal evolution under immunotherapy pressure: primary intratumoral heterogeneity already harbors multiple distinct resistant subclones at initial diagnosis, while continuous PD-1 blockade further selects out immune-evasive clones to drive acquired drug resistance, creating spatially separated lesion regions with divergent resistance pathways within a single tumor mass. Regulatory T cells (Tregs) are frequently enriched in the melanoma microenvironment and suppress anti-tumour immunity by secreting IL-10, IL-35 and TGF-β and by expressing inhibitory receptors such as CTLA-4, PD-1 and LAG-3. The intratumoral CD8^+^ T-cell/Treg ratio is associated with patient survival in melanoma (Huang et al., 2021).

High expression of immunosuppressive molecules further aggravates the immunosuppressive state of the TME. In a cohort of 94 melanoma tumors sampled at baseline and at PD-1 inhibitor progression, MHC class I downregulation was a hallmark of resistance to PD-1 inhibitors and was associated with cancer-associated fibroblast signatures and the MITF^low^/AXL^high^ de-differentiated phenotype (Lee et al., 2020). TGF-β drove this treatment-resistant phenotype and contributed to MHC class I downregulation in melanoma, indicating that stromal TGF-β/CAF programs are linked to—rather than sole determinants of—resistance to PD-1/PD-L1 blockade (Lee et al., 2020). High expression of PD-L1 on the surface of tumor cells and immunosuppressive cells can directly bind to PD-1 on the surface of T cells, inhibiting T cell activation and proliferation (Tumeh et al., 2014). Overexpression of indoleamine 2,3-dioxygenase (IDO1) catalyses tryptophan catabolism to produce kynurenine. Ultimately, the building up of kynurenine drives suppression of effector T-cell function and consequently promotes resistance to PD-1 inhibitors (Holmgaard et al., 2013). After being activated by IFN-γ, the NLRP3 inflammasome inhibits cytotoxic T cells by recruiting MDSCs, mediating resistance to PD-1 inhibitors in melanoma (Theivanthiran et al., 2020).

Acidic microenvironment and extracellular matrix barriers are also involved in the formation of drug resistance. Abnormally enhanced glycolytic metabolism enables tumor cells to compete with effector T cells for glucose—a mechanism first characterized in cancer models and subsequently invoked in melanoma (Chang et al., 2015). Accumulation of lactic acid in the TME inhibits CD8^+^ T cell activation and promotes M2 macrophage polarization, as demonstrated across solid-tumor models and later extrapolated to the melanoma TME (Wang et al., 2020). During therapy-tolerant residual disease, collagen-rich extracellular matrix remodeling forms a physical barrier that spatially excludes CD8^+^ T cells from residual tumor niches, restricts immune infiltration, and contributes to diminished responsiveness to immunotherapy in melanoma (Hsu et al., 2025).

The above TME-mediated immunosuppression involves the combined actions of various cells, molecules and physical barriers. The core components and drug resistance regulatory functions are summarized in Table 2. The metabolic microenvironment disorder in TME is closely related to the intrinsic metabolic reprogramming of tumor cells, which is further analyzed in Section 2.3.

**TABLE 2:** Immunosuppressive components of the TME in melanoma and their contribution to PD-1/PD-L1 inhibitor resistance.

TME component	Key mediators	Immunosuppressive mechanism(s)	Therapeutic strategies	Evidence
Myeloid-derived suppressor cells (MDSCs)	PGE2, G-CSF, GM-CSF, CCR5; iNOS, ROS, Arginase-1	Inhibit T cell activation; induce T cell apoptosis	STAT3 inhibitors (napabucasin); MDSC-targeted depletion	Clinical validation
M2-polarized tumor-associated macrophages (TAMs)	IL-10, TGF-β; TAM nanocomplex targets	Suppress CD8^+^ T cells; promote immunosuppression	TAM-targeted nanocomplexes; MARCO targeting; rewiring to M1	Preclinical
Regulatory T cells (Tregs)	IL-10, IL-35, TGF-β; LAG-3, PD-1, CTLA-4	Suppress effector T cells; inhibit anti-tumor immunity	Anti-CTLA-4; anti-LAG-3; low-dose radiotherapy	Established
Cancer-associated fibroblasts (CAFs)	CXCL12, CXCL14, IL-6; TGF-β	Collagen barrier; TGF-β activation; T-cell exclusion	TGF-β pathway inhibitors; CAF-depleting agents	Preclinical
Dendritic cells (DCs, dysfunctional)	Reduced antigen presentation; tolerogenic phenotype	Fail to prime CD8^+^ T cells	TLR7/8/RIG-I agonists (CV8102); DC vaccines	Clinical validation
Lactic acid/acidic microenvironment	Lactate; H+ accumulation	Inhibits CD8^+^ T cells; promotes M2 polarization; upregulates PD-1 on Tregs	Glycolysis inhibitors; lactate transporters	Preclinical
Dense/remodeled ECM in residual melanoma	Collagen I/III, elastin, laminin, TGFB2 (ECM-related genes induced during residual disease)	Physical barrier; spatially excludes CD8^+^ T cells from residual tumor niches; impairs immune surveillance and contributes to therapy tolerance/resistance	Pharmacologic inhibition of collagen deposition (P4HA inhibitor DHB) or collagen crosslinking (LOX inhibitor BAPN); ECM modulation during the residual-disease window	Immune-competent BRAF-mutant melanoma model; human melanoma datasets
IDO1 overexpression	Tryptophan→kynurenine	Tryptophan depletion coupled with kynurenine accumulation; kynurenine buildup impairs effector T-cell function and mediates immunosuppression	IDO1 inhibitors (combined with PD-1 inhibitors)	Clinical validation
NLRP3 inflammasome (tumor-intrinsic)	IFN-γ-activated NLRP3	Recruits MDSCs; inhibits cytotoxic T cells	NLRP3 modulators (context-dependent)	Preclinical
CAFs/TGF-β	CAF TGF-β signature; TGF-β ligands	Associated with MITF^low^/AXL^high^ dedifferentiation and MHC-I downregulation; linked to PD-1/PD-L1 blockade resistance	Anti-TGF-β antibodies; TGF-βRI inhibitors	Clinical validation

Evidence levels: Evidence grading standards are consistent with Table 1. Abbreviations: PGE2, prostaglandin E2; G-CSF, granulocyte colony-stimulating factor; GM-CSF, granulocyte-macrophage colony-stimulating factor; CCR5, C-C motif chemokine receptor 5; iNOS, inducible nitric oxide synthase; ROS, reactive oxygen species; STAT3, signal transducer and activator of transcription 3; IL, interleukin; TGF-β, transforming growth factor beta; MARCO, macrophage receptor with collagenous structure; LAG-3, lymphocyte activation gene 3; CTLA-4, cytotoxic T-lymphocyte-associated protein 4; CXCL, C-X-C motif chemokine ligand; CAFs, cancer-associated fibroblasts; TLR, Toll-like receptor; RIG-I, Retinoic acid–inducible gene I; IDO1, indoleamine 2,3-dioxygenase; NLRP3, NLR, family pyrin domain containing 3; MDSCs, myeloid-derived suppressor cells.

### 2.3 Metabolic reprogramming mechanism

Tumor cell metabolic reprogramming participates in the formation of resistance to PD-1/PD-L1 inhibitors by regulating the energy state and functional activity of tumor cells and immune cells, mainly manifested as enhanced glycolysis, lactic acid accumulation, abnormal fatty acid metabolism and imbalance of trace element metabolism.

TEAD3^+^ high-risk melanoma cells crosstalk with GAS6^+^ macrophages via the GAS6-TYRO3 ligand-receptor axis to modulate propionate metabolism and drive melanoma progression. Fang et al. revealed that this metabolic interplay supports tumor cell survival and suppresses CD8^+^ T cell function, representing a novel stromal-tumor metabolic axis contributing to resistance (Fang et al., 2025). Proteomics analyses of melanoma response to immunotherapy reveal mitochondrial dependence as a key metabolic feature associated with resistance (Harel et al., 2019). Copper availability supports oncogenic MAPK signaling in BRAF^v600E^ melanoma. Copper import through CTR1 enables MEK1/2 kinase activity, and pharmacologic copper chelation with tetrathiomolybdate (TTM) suppresses MAPK signaling, inhibits the growth of melanoma cells bearing MAPK-dependent resistance drivers or acquired BRAF/MEK-inhibitor resistance, and synergizes with BRAF/MEK inhibitors in BRAF^v600E^ melanoma models (Brady et al., 2017). The dual role of copper metabolism in targeted drug resistance and immune resistance makes it a potential pharmacological target for combination therapy, and related intervention strategies are detailed in Section 3.2.2.

### 2.4 Epigenetic reprogramming mechanism

Abnormal epigenetic modification participates in the formation of drug resistance by regulating gene expression, and is an important regulatory layer connecting external immune stimulation and tumor cell drug resistance phenotype.

Across multiple tumor types, cells resistant to immune checkpoint blockade acquire interferon-associated epigenetic memory that sustains T-cell dysfunction; whether this exact program dominates in melanoma remains to be defined (Qiu et al., 2023). Overexpression of histone deacetylase (HDAC) inhibits IFN-γ-induced MHC-II expression through chromatin remodeling, mediating resistance to PD-1 inhibitors in melanoma (James et al., 2023). Enhancer of zeste homolog 2 (EZH2) (PRC2 complex) inhibits IFN-γ-induced MHC-II expression through chromatin remodeling, mediating immune resistance in melanoma (James et al., 2023). Hypermethylation of the STING promoter leads to silencing of the cyclic GMP-AMP synthase-stimulator of interferon genes (cGAS-STING) pathway, and melanoma maintains a “cold” phenotype and is resistant to immunotherapy (Mah et al., 2024). As an epigenetic effector molecule, PARP14 shapes chromatin states via the recruitment of histone deacetylase complexes. Within the epigenetic regulatory landscape, this process suppresses the transcriptional output of IFN-γ-responsive gene sets. Such epigenetic repression constitutes one layer of adaptive tumour resistance that emerges under sustained IFN-γ stimulation, ultimately blunting therapeutic responses to PD-1 inhibitors (Wong et al., 2023). The reversibility of epigenetic regulation makes it an important pharmacological intervention target, and corresponding reversal strategies are detailed in Section 3.2.3.

### 2.5 Systemic regulatory mechanism

Systemic factors indirectly affect the sensitivity of melanoma to PD-1/PD-L1 inhibitors by regulating the overall immune state of the body and the TME, among which intestinal flora disorder is the core systemic regulatory factor.

The composition and diversity of intestinal flora directly determine the clinical response of melanoma patients to anti-PD-1 immunotherapy (Gopalakrishnan et al., 2018). Patients with drug resistance have enriched harmful flora such as Clostridiaceae in the intestine, and lack beneficial flora such as Ruminococcaceae and Bifidobacteriaceae, forming an immunosuppressive intestinal microenvironment (Simiczyjew et al., 2020). Intestinal flora disorder can inhibit systemic anti-tumor immunity, downregulate the activation of IFN-γ pathway, reduce the function of effector T cells in the TME, and aggravate resistance to PD-1 blockade (Baruch et al., 2021). Fecal microbiota transplantation (FMT) can reshape the host immune microenvironment and reverse the resistance to ICIs in patients with advanced melanoma, providing direct evidence for intestinal flora-mediated resistance (Davar et al., 2021). In preclinical tumor models, microbiome-derived inosine modulates response to checkpoint inhibitor immunotherapy, providing a conceptual basis that may also operate in melanoma (Mager et al., 2020). Metabolites of intestinal flora can remotely regulate the TME through blood circulation, enhancing or inhibiting the anti-tumor effect of immune checkpoint inhibitors (Davar et al., 2021). However, clinical translation of FMT is currently limited by heterogeneous donor selection criteria and a paucity of long-term safety data (Simiczyjew et al., 2020).

In addition, elevated baseline plasma IL-6 levels are closely related to resistance to immune checkpoint blockers in melanoma patients (Wang Y. et al., 2022). Serum β2-microglobulin level can be used as a prognostic indicator of melanoma progression, indirectly reflecting tumor burden and immune state, and its abnormal elevation may indicate the risk of drug resistance (Wang C. et al., 2022).

### 2.6 Combinatorial anti-tumor effects of therapeutic strategies against PD-1/PD-L1 inhibitor resistance

The above-mentioned resistance mechanisms at all levels do not act independently, but have close cross-regulation and combinatorial therapeutic effects. Defects in tumor cell antigen presentation lead to insufficient infiltration of CD8^+^ T cells, further aggravating TME immunosuppression (Sade-Feldman et al., 2017); while inhibitory cytokines secreted by immunosuppressive cells in the TME can promote tumor cell phenotypic transformation and activation of resistance-related signaling pathways (Pozniak et al., 2024; Kim et al., 2021). Metabolic reprogramming not only directly affects the proliferation and survival of tumor cells, but also participates in the formation of drug resistance by regulating immune cell function: glucose competition and lactic acid accumulation caused by enhanced tumor cell glycolysis directly inhibit CD8^+^ T cell function (Chang et al., 2015; Wang et al., 2020). Epigenetic modification can simultaneously regulate tumor cell antigen expression, metabolic enzyme activity and microenvironment cell polarization, serving as a critical regulatory hub connecting multi-dimensional mechanisms (Pozniak et al., 2024; James et al., 2023; Wong et al., 2023). Notably, this combinatorial network exhibits subtype-specific divergence: uveal melanoma rarely harbors B2M loss and relies heavily on TME TGF-β-mediated immunosuppression; while acral melanoma prioritizes epigenetic silencing pathways over MAPK-driven immune escape, creating divergent dominant resistance axes across melanoma subgroups (Ma et al., 2025). Systemic factors such as intestinal flora disorder indirectly affect the TME by regulating systemic immune state, forming a remote regulatory axis of “intestine-immunity-tumor” (Simiczyjew et al., 2020). The preceding sections delineate four functionally distinct yet highly interactive layers mediating PD-1/PD-L1 inhibitor resistance in melanoma. Rather than operating in isolation, these axes engage in bidirectional crosstalk—such as lactate-mediated immunosuppression and cytokine-driven epigenetic remodeling—to form a combinatorial regulatory network. These complex interactions are visualized in Figure 2. This schematic explicitly incorporates clinical translational anchors, linking specific resistance mechanisms to actionable biomarkers (yellow tags) and corresponding therapeutic vulnerabilities (blue tags), while contextualizing these interactions within the backdrop of intratumoral clonal heterogeneity. Distinct mechanistic weighting exists between primary and acquired melanoma resistance driven by this multidimensional network. Primary resistance predominantly originates from inherent tumor defects including germline or early somatic B2M/MAPK mutations and baseline immunosuppressive gut microbiota composition, while acquired resistance evolves via epigenetic memory remodeling, lactate-mediated persistent TME suppression and gradual clonal selection under long-term ICI exposure. Biomarker screening and combinational therapeutic design should differentiate these two resistance subtypes to achieve more precise intervention.

**FIGURE 2:**
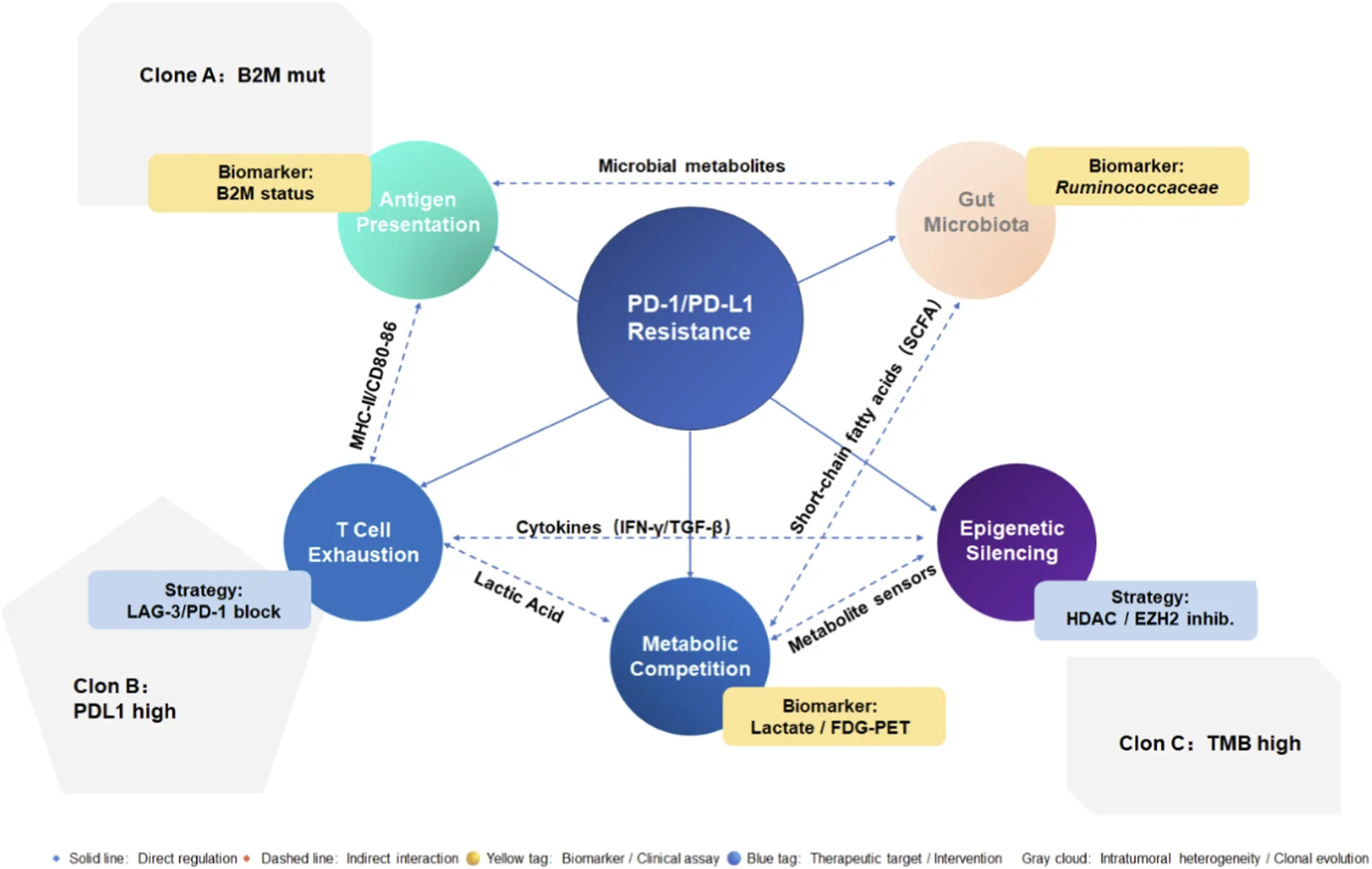
Overview of multidimensional resistance mechanisms underlying PD-1/PD-L1 inhibitor therapy in melanoma. The figure illustrates tumor-intrinsic defects, immunosuppressive microenvironment remodeling, metabolic rewiring, epigenetic regulation, and systemic immune modulation, as well as their bidirectional crosstalk during acquired resistance to immune checkpoint blockade. Abbreviations: PD-1, programmed cell death protein 1; PD-L1, programmed death-ligand 1; CTLA-4, cytotoxic T-lymphocyte-associated protein 4; MHC-I, major histocompatibility complex class I; MHC-II, major histocompatibility complex class II; TCR, T cell receptor; IFN-γ, interferon-gamma; TMB, tumor mutational burden; TME, tumor microenvironment; ICI, immune checkpoint inhibitor.

The central hub denotes the resultant drug resistance phenotype. Surrounding nodes represent five core regulatory axes: (1) Antigen Presentation Defect (Biomarker: *B2M* mutational status), (2) T Cell Exhaustion (Intervention: LAG-3/PD-1 dual blockade), (3) Metabolic Competition (Biomarker: lactate levels/FDG-PET avidity), (4) Epigenetic Silencing (Intervention: HDAC/EZH2 inhibition), and (5) Systemic Regulation by Gut Microbiota (Biomarker: Ruminococcaceae abundance). Solid blue lines depict direct mechanistic contributions to the resistance phenotype. Blue dashed lines illustrate bidirectional crosstalk among axes, annotated with key mediating molecules (e.g., lactic acid shuttling, cytokine signaling, or microbial metabolites). Light gray clouds at the base signify intratumoral clonal heterogeneity, harboring distinct genetic alterations such as *B2M* loss, *PD-L1* amplification, or high tumor mutational burden (TMB). Yellow and blue tags denote clinically actionable biomarkers and corresponding therapeutic vulnerabilities, respectively. This architecture highlights that resistance is not governed by linear pathways but by a multifactorial interactive network, aligning with recent spatial ecology findings as described below.

## 3 Strategies to reverse resistance to PD-1/PD-L1 inhibitors in melanoma

Aiming at the multi-dimensional mechanisms of PD-1/PD-L1 inhibitor resistance in melanoma, a series of targeted reversal strategies have been verified in preclinical or clinical studies. Immune combination regimens primarily target the immunosuppressive status of the TME and T cell exhaustion; targeted combinations address intrinsic pathway abnormalities, metabolic rewiring, and epigenetic reprogramming; cellular and local therapies cover refractory scenarios such as antigen presentation defects.

### 3.1 Immune combination regimens

Dual immune checkpoint blockade is achieved by engaging distinct immune regulatory pathways to improve treatment outcomes. Phase III evidence confirms that CTLA-4 plus PD-1 inhibition yields superior survival over monotherapy in advanced melanoma and can reverse partial PD-1 monotherapy resistance, albeit with substantial toxicity—grade 3–4 treatment-related adverse events occur in 55% of patients receiving the combination (Larkin et al., 2015). The LAG-3 inhibitor relatlimab combined with nivolumab (Opdualag) inhibits CD4+/CD8+ T cell exhaustion and expands the population benefiting from immunotherapy, providing a new option for PD-1-resistant patients (Tawbi et al., 2022); 3-year follow-up further supports durable benefit of the nivolumab-plus-relatlimab combination in advanced melanoma (Tawbi et al., 2024). Mechanistically, LAG3 and PD-1 cooperate on CD8^+^ T cells to drive exhaustion and hinder autocrine IFNγ-dependent anti-tumor immunity (Andrews et al., 2024). Targeting TIGIT represents another axis: in melanoma, the TIGIT-CD155 (also termed PVR) axis suppresses cytotoxic T-cell function and contributes to both primary and acquired resistance against PD-1 inhibitors (Kawashima et al., 2021). Combined PD-1 and TIM-3 blockade in the AMBER trial cohorts 1c, 1e, and 2A demonstrated clinical activity in locally advanced or metastatic melanoma (Davar et al., 2025).

Oncolytic virotherapy reverses resistance through direct oncolysis and immune activation. Oncolytic viruses lyse tumor cells, release tumor antigens and damage-associated molecular patterns (DAMPs), activate dendritic cells, promote CD8^+^ effector T-cell infiltration, and reduce Tregs, MDSCs, and M2 macrophages in the TME (Ribas et al., 2017). A phase 1b trial (Ribas et al., 2017) enrolling 21 patients with advanced melanoma treated with talimogene laherparepvec followed by pembrolizumab reported a confirmed objective response rate of 61.9% and a complete-response rate of 33.3% per immune-related response criteria. Treatment was generally well-tolerated without dose-limiting toxicities. Responding patients demonstrated increased intratumoral CD8^+^ T-cell infiltration, elevated PD-L1 expression, and enhanced IFN-γ transcriptional signatures after talimogene laherparepvec administration, indicating that oncolytic virotherapy remodels the TME to potentiate anti-PD-1 activity (Ribas et al., 2017). Findings originating from the global phase III MASTERKEY-265 trial demonstrated superior objective response rates for the combination of talimogene laherparepvec (T-VEC) and pembrolizumab relative to pembrolizumab monotherapy (48.6% versus 41.3%), alongside elevated complete-response rates. This regimen can counteract PD-L1-driven therapeutic resistance within advanced-melanoma patient cohorts (Chesney et al., 2023).

Intratumoral CV8102, a TLR7/8 and RIG-I agonist, showed a manageable safety profile in a phase I study enrolling advanced melanoma and other solid tumor patients (Eigentler et al., 2025). RP1 (vusolimogene mwaenigenia) combined with nivolumab achieved an objective response rate (ORR) of 32.9% (15.0% CR) in PD-1-resistant melanoma, with a median duration of response of 33.7 months (95% CI: 14.1 to not reached; data cutoff: 8 March 2024); among 46 responding patients, 96.6% of non-injected lesions demonstrated any reduction, indicating a systemic immune-mediated antitumor effect (Wong et al., 2025).

Neoantigen vaccines overcome antigen-presentation-defect-mediated resistance by activating tumor-specific T cells. Personalized neoantigen vaccines can precisely activate tumor-specific CD4^+^ and CD8^+^ T cells, enhance tumor immunogenicity, and improves response to PD-1 inhibitors (Ott et al., 2017). HDL-mimicking nanodisks markedly improve neoantigen delivery in preclinical platforms; in a mouse colon-cancer model, nanovaccine plus PD-1 antibody achieved ∼88% complete regression, illustrating a delivery principle that can be further tested in melanoma cancer models (Kuai et al., 2017). Allogeneic plasmacytoid dendritic cell vaccines can break immune tolerance by priming and expanding specific CD8^+^ T cells; *in vitro* studies confirm combinatorial therapeutic effects with anti-PD-1 antibody (Plumas, 2022).

Despite accumulating mechanistic insights from pre-clinical and early-phase clinical studies, several notable limitations remain to be addressed. Most mechanistic evidence originates from pre-clinical animal models (Yan et al., 2021; Nakamura et al., 2021). These experimental systems cannot fully recapitulate human melanoma heterogeneity, TME complexity, patient-specific immune background, and genetic diversity, forming a substantial translational gap between bench discoveries and clinical practice. Many combinatorial strategies that exhibit promising anti-tumor activity in mouse models frequently fail to achieve consistent therapeutic benefits in human patients. In addition, most available clinical data for resistance-reversal regimens rely on small-size, single-arm early-phase trials. Large-scale randomized controlled clinical evidence is still lacking. Further efforts are urgently required to bridge pre-clinical mechanistic findings and real-world clinical outcomes.

### 3.2 Targeted combination regimens

Targeted combinations reverse resistance by inhibiting resistance-related pathways, regulating metabolism or epigenetic state, and cooperating with PD-1 inhibitors to achieve therapeutic intervention.

#### 3.2.1 Combinations targeting signaling pathways

The triple regimen of BRAF and MEK inhibitors plus a PD-1 inhibitor improves the immunosuppressive microenvironment by increasing CD8^+^ T-cell infiltration, upregulating MHC class I/II antigen-presentation machinery, and enhancing T-cell-inflammatory gene signatures, thereby producing durable responses and partially overcoming acquired resistance in BRAF-mutant advanced melanoma (Ribas et al., 2019). From a mechanistic standpoint, MAPK/ERK pathway inhibition reinstates tumor antigen expression and creates a transient “immune-permissive” window with enhanced CD8^+^ infiltration, although it concurrently induces adaptive PD-L1 and T-cell-exhaustion markers—a bimodal remodeling that rationalizes timed PD-1 inhibitor addition (Meng et al., 2026). Extending beyond BRAF-mutant disease, combined RAS/MEK/PI3K inhibition with a CD40 agonist upregulates CD40 on melanoma cells and selectively suppresses CD11b^+^ regulatory B-cell (Breg) expansion, reversing PD-1 resistance and driving near-complete regression in NRAS-mutant melanoma models (Yan et al., 2026).

For STING pathway dysfunction, lipid nanoparticle delivery of STING agonists effectively activates NK cells and overcomes anti-PD-1 resistance in a melanoma lung metastasis model (Nakamura et al., 2021). PARP14 inhibitors restore sensitivity of chronic IFN-γ-induced drug-resistant tumors to PD-1 blockade, inhibiting resistant melanoma growth and prolonging mouse survival (Wong et al., 2023).

#### 3.2.2 Combinations targeting metabolic pathways

Although MAPK/ERK inhibition creates a transient immune-permissive window, acquired resistance ultimately consolidates an immune-evasive microenvironment, arguing for layering metabolic and cell-death-targeted regimens atop signaling blockade (Meng et al., 2026). Copper-targeted interventions act on two separable therapeutic axes in melanoma: copper chelators reduce intracellular copper, inhibit the MAPK pathway, and reverse BRAFi/MEKi resistance; copper ionophores such as elesclomol form Cu^2+^ complexes that are delivered to mitochondria, increase ROS, and are preferentially cytotoxic to OXPHOS-dependent melanoma cells—including vemurafenib-resistant and JARID1B-high subpopulations—with copper-dependent cell death (cuproptosis) most relevant in OXPHOS-proficient melanoma models (Chrzan and Hartman, 2025). Repurposed disulfiram (DSF) is metabolized to diethyldithiocarbamate (DDTC), binds Cu^2+^, induces ROS/apoptosis/ferroptosis/cuproptosis, and sensitizes MEK inhibitors and PD-1 inhibitors (Meraz-Torres et al., 2024). Succinate dehydrogenase (SDH) inhibitors (DMM, 3-NPA) cause succinate accumulation and elevated succinyl-CoA, inducing PD-L1 succinylation at lysine-129 (K129) that promotes lysosomal-dependent degradation and enhances CD8^+^ T-cell-mediated tumour-cell killing (Liang et al., 2025). This post-translational modification, catalysed by carnitine palmitoyltransferase 1A (CPT1A), does not alter PD-L1 transcript abundance, but shortens PD-L1 protein half-life via endosome-lysosomal clearance, reducing PD-L1 abundance on the melanoma cell surface. Reduced surface PD-L1 blunts PD-1-dependent inhibitory signalling and reinvigorates CD8^+^-T-cell-dependent anti-tumour immunity. Consistently, the non-succinylatable K129R mutation protects PD-L1 from lysosomal turnover and restores PD-L1-driven immune evasion (Liang et al., 2025).

#### 3.2.3 Combinations targeting epigenetic pathways

HDAC inhibitors upregulate immune checkpoint ligands such as PD-L1 and, combined with PD-1 inhibitors, significantly enhance anti-tumor efficacy in melanoma (Woods et al., 2015). METTL3 deletion stabilizes the IFN-γ/STAT1/IRF1 pathway and enhances melanoma sensitivity to anti-PD-1 therapy (Wu et al., 2024).

### 3.3 Cellular and local therapies

Adoptive cell therapy (ACT) remains a conceptually distinct route to overcome PD-1-refractory melanoma. The autologous tumor-infiltrating lymphocyte (TIL) product lifileucel, evaluated in the C-144-01 single-arm phase II trial, produced an objective response in 36% of heavily pretreated metastatic melanoma patients, rising to 41% in the subgroup primarily refractory to anti-PD-1 or PD-L1 blockade (Sarnaik et al., 2021). Beyond bulk TIL, neoantigen-directed and antigen-selected ACT have been framed within personalized melanoma care, where TIL therapy exemplified by lifileucel already demonstrates activity in BRAF-mutant disease (Shebrain et al., 2024). In broader solid-tumor frameworks, T-cell receptor (TCR)-engineered T cells targeting intracellular neoantigens offer a route to bypass antigen-presentation defects such as B2M loss; translation to melanoma is ongoing but not yet established by the cited reference (Wang et al., 2021). In B2M-deficient tumors, endogenous NK cells cooperating with CD4^+^ T cells can sustain anti-tumor surveillance independently of CD8^+^ recognition (Torrejon et al., 2023); In the B16-OVA-hCD19 melanoma model, CAR-NKTs controlled tumor growth more effectively than CAR-Ts and prolonged survival without evident toxicity, eliminating CD1d-expressing M2-like macrophages and promoting epitope spreading; under high tumor burden, however, they acquired PD-1/TIM-3-mediated exhaustion that was reversed by PD-1 blockade or vaccination, and clinical validation is still pending (Zhou et al., 2024). The broader translation of ACT is still bounded by practical constraints: leukapheresis and *ex vivo* TIL expansion is costly and labor-intensive, lymphodepletion pre-conditioning carries hematologic toxicity in older or frail patients, and TCR-T products remain HLA-restricted (Sarnaik et al., 2021; Wang et al., 2021).

Local modalities intersect with checkpoint blockade through distinct immune layers. In B16 melanoma, hypofractionated radiotherapy paired with low-dose cyclophosphamide and PD-L1 blockade reduces intratumoral Tregs and expands CD8^+^ effector infiltration at both irradiated and distant lesions, producing an abscopal response (Luo and Zeng, 2024); hypofractionated radiation together with PD-1/PD-L1 inhibition additionally engages the cGAS-STING axis and DAMP release to convert immune-deserted tumors into infiltrated ones (Twyman-Saint Victor et al., 2015). Combining radiotherapy with anti-CTLA-4 depletes Tregs and enriches CD4^+^ effector T cells, and adding anti-PD-1/PD-L1 raises complete regression of unirradiated lesions to roughly 80% in murine melanoma (Twyman-Saint Victor et al., 2015). Radiation with dual checkpoint blockade mobilizes non-redundant innate and adaptive arms rather than simply adding their effects (Twyman-Saint Victor et al., 2015).

Sonodynamic strategies extend local immune priming into melanoma models. CuO_2_ nanosonosensitizers delivered in a thermosensitive hydrogel generate Fenton and sonodynamic reactive ROS in B16 tumors, amplifying oxidative stress and promoting immunogenic cell death with consequent dendritic cell maturation and CD8^+^ recruitment (Zheng et al., 2023). Chemotherapeutic agents can similarly act as immunogenic cell death (ICD) inducers in melanoma: chromomycin A5 exposes calreticulin, releases ATP and HMGB1, and activates antigen-presenting cells and T lymphocytes in B16-F10 models, recapitulating the adjuvant effects ascribed to anthracyclines or oxaliplatin in other settings (Dias Florêncio et al., 2022). These observations place ICD-based local or systemic priming as a plausible companion to PD-1 blockade in melanoma, though clinical confirmation is still pending. The aforementioned reversal strategies, including immune-based combinations, targeted regimens, cellular therapies and local intervention approaches, are summarized in Table 3.

**TABLE 3:** Resistance reversal strategies for PD-1/PD-L1 inhibitor failure in melanoma: evidence hierarchy, efficacy, toxicity, and clinical applicability.

Strategy (category)	Target/mechanism	Evidence level [supporting reference]	Key efficacy data	Toxicity profile	Patient selection/biomarker	Key limitations/challenges
Immune combination regimens						
Dual ICI (Nivo + Ipi) (immune combo)	CTLA-4 + PD-1	Established (Ph III) (Larkin et al., 2015)	CheckMate 067; 5 years OS 52%; EFS superior to monotherapy	Gr 3–4 TRAE 55% (primary) → 59% (5 years); irAEs common	PD-L1, TMB, CD8+/Treg; exclude autoimmunity	High toxicity in unfit/elderly; long term irAE mgmt
Nivo + Relatlimab (Opdualag) (immune combo)	PD-1 + LAG-3	Established (Ph III) (Tawbi et al., 2022; Tawbi et al., 2024)	RELATIVITY-047: mPFS 10.1 vs. 4.6 months (HR 0.75); 3 years OS data mature	Gr 3–4 TRAE lower than Nivo + Ipi	CD8^+^ T cell exhaustion signature	Modest HR; biomarker for responder subset needed
PD-1 + TIM-3 (AMBER) (immune combo)	PD-1 + TIM-3	Clinical validation (Davar et al., 2025)	AMBER 1c/1e/2A: clinical activity in advanced/metastatic melanoma	Manageable; still being characterized	TIM-3+ CD8^+^ T cell infiltrate	Early phase; randomized data pending
Oncolytic based combination therapies						
T-VEC + Pembrolizumab (oncolytic + ICI)	Oncolytic HSV-1 + PD-1	Clinical validation (Ph 1b) (Ribas et al., 2017); Established (Ph III) (Chesney et al., 2023)	Phase1b: ORR 61.9%, CR 33.3%; MASTERKEY-265: ORR 48.6% vs. 41.3% (mono); improved CR	Flu-like, injection site; Gr 3–4 rare	Injectable lesions + PD-L1 high	Requires injectable disease; cold tumor conversion variable
CV8102 + ICI (oncolytic + ICI)	TLR7/8 + RIG-I agonist	Clinical validation (Eigentler et al., 2025)	Phase I: manageable safety in melanoma and other solid tumors	Mild-moderate; intratumoral route	Injectable lesions	Phase I; efficacy data immature
RP1 + Nivolumab (IGNYTE) (oncolytic + ICI)	HSV-1 RP1 + PD-1	Clinical validation (Ph II) (Wong et al., 2025)	ORR 32.9% (15% CR); mDOR 33.7 months; 96.6% non-injected lesions regressed	Gr ≥ 3 TRAE 12.9%	Anti-PD-1 failed melanoma	Single arm; FDA CRL 2025; randomized trial needed
Targeted signaling pathway combinations						
BRAF/MEK inhibitors + PD-1 inhibitors (targeted + ICI)	BRAF + MEK + PD-1	Established (Ph III) (Ribas et al., 2019)	IMspire150/KEYNOTE; IMspire151; long term responders in BRAF mutant	Overlapping toxicity (pyrexia, hepatotoxicity)	BRAF V600E/K; CD8^+^ T cell infiltrate	Sequencing debate; triplet vs. sequential
RAS/MEK/PI3K inhibitors + CD40 agonist (targeted + ICI)	MAPK pathway + CD40	Preclinical (Yan et al., 2026)	∼82.9% tumor growth inhibition, 44% CR (4/9) in PD-1-resistant NRASmut B16 melanoma	Not yet characterized *in vivo*	NRAS mutant; CD11b+Breg signature	Preclinical; human translation pending
STING agonists (LNP) (targeted innate)	STING pathway	Preclinical (Nakamura et al., 2021)	Overcomes anti-PD-1 resistance in lung mets (mouse)	Systemic inflammation risk	STING-low/STING-methylated tumors	Delivery and systemic toxicity
Metabolic targeted therapeutic strategies						
Copper-targeted therapy (metabolic targeted)	Copper chelation/ionophore	Preclinical (Chrzan and Hartman, 2025; Meraz-Torres et al., 2024)	Reverses BRAFi/MEKi resistance; induces cuproptosis	Systemic Cu homeostasis disruption	BRAF mutant high OXPHOS	Therapeutic window undefined
SDH inhib (DMM/3-NPA) (metabolic/epigenetic)	Succinate dehydrogenase	Preclinical (Liang et al., 2025)	PD-L1 succinylation → lysosomal degradation; enhances CD8^+^ kill	Metabolic perturbations	Tumors with high succinate/SDH dysregulation	Preclinical only
Epigenetic targeted combinations						
PARP14 inhibitors (epigenetic)	PARP14 chromatin remodeling	Preclinical (Wong et al., 2023)	Restores IFNγ sensitivity in resistant tumors (mouse)	Under investigation	IFNγ-driven resistant tumors	Preclinical; specificity concerns
HDAC inhibitors + PD-1 inhibitors (epigenetic)	HDAC/MHC-II	Preclinical/early clinical (Woods et al., 2015)	Upregulates PD-L1 & MHC-II; enhances anti-tumor efficacy	Myelosuppression; fatigue	MHC-II low/PRC2-high tumors	Paradoxical PD-L1 upregulation
METTL3 modulators (epigenetic)	m6A RNA methylation	Preclinical (Wu et al., 2024)	Stabilizes IFNγ/STAT1/IRF1; enhances PD-1 sensitivity	Off-target RNA effects	m6A low tumors	Tool compounds; *in vivo* validation needed
Cellular adoptive therapies						
TIL (Lifileucel) (cellular)	Adoptive T cell transfer	Established (Ph II) (Sarnaik et al., 2021)	ORR 36% (41% in PD-1-primary-refractory subset), C-144-01 trial	Lymphodepletion + IL-2: cytopenia, CRS	Harvestable TIL	Tumor harvesting; mfg cost; conditioning toxicity
TCR-T therapy (cellular)	TCR-engineered T cells	Preclinical (Wang et al., 2021)	IL-7 culture without CD3 activation enables efficient TIL1383I TCR transduction; TN/TSCM-enriched product with longer telomeres and superior *in vivo* anti-melanoma response	On-target/off-tumor; CRS	B2M-deficient; HLA-matched	HLA restriction; safety
NK/CAR-iNKT cell therapy (cellular)	Innate lymphoid cells	Preclinical/early clinical (Torrejon et al., 2023)	B2M-def: NK + CD4 substitute	CRS; NK persistence	B2M-deficient tumors	Early stage; persistence and scale-up
Local intervention strategies						
HFRT + low-dose cyclophosphamide + anti-PD-L1 (local)	Treg depletion/CD8 activation/abscopal	Preclinical (Luo and Zeng, 2024)	Bilateral B16: Treg↓, CD8↑, abscopal with triple combo	LD-CYC myelosupp; RT skin tox	Irradiable + distal lesions	Optimal LD-CYC dose/fractionation undefined; not pure RT
RT + dual ICI (local + ICI)	Treg↓/CD4eff↑/non-redundant	Clinical validation (Twyman-Saint Victor et al., 2015)	∼80% CR of unirradiated lesions in B16 (RT + anti-CTLA4+anti-PD1)	Combined irAE	Oligometastatic; avoid steroids	Steroid use worsens survival (HR 1.56)
Vaccine based immunotherapies						
Personalized neoantigen vaccine + PD-1 inhibitors (vaccine)	Neoantigen-specific CD4/CD8 T cells	Clinical validation (Ph I) (Ott et al., 2017)	Durable responses in high-risk resected melanoma	Mild-moderate; injection site	Sufficient TMB for neoantigen prediction	Manufacturing time and cost
Allogeneic pDC vaccine (vaccine)	Plasmacytoid DC priming	Preclinical/early clinical (Plumas, 2022)	Breaks tolerance; synergizes with anti-PD-1	Under investigation	Tolerogenic DC phenotype	Early stage

Evidence levels: Evidence grading standards are consistent with Table 1. Abbreviations: ICI, immune checkpoint inhibitor; Nivo, nivolumab; Ipi, ipilimumab; LAG-3, lymphocyte-activation gene 3; TIM-3, T-cell immunoglobulin and mucin-domain containing-3; HSV, herpes simplex virus; BRAF, v-raf murine sarcoma viral oncogene homolog B; MEK, mitogen-activated protein kinase kinase; MAPK, mitogen-activated protein kinase; PI3K, phosphoinositide 3-kinase; STING, stimulator of interferon genes; LNP, lipid nanoparticle; Cu, copper; SDH, succinate dehydrogenase; PARP, poly(ADP-ribose) polymerase; HDAC, histone deacetylase; MHC, major histocompatibility complex; PRC2, polycomb repressive complex 2; m6A, N6-methyladenosine; IFNγ, interferon gamma; METTL3, methyltransferase-like 3; STAT1, signal transducer and activator of transcription 1; IRF1, interferon regulatory factor 1; TIL, tumor-infiltrating lymphocyte; TCR, T-cell receptor; B2M, beta-2-microglobulin; NK, natural killer; iNKT, invariant natural killer T; CRS, cytokine release syndrome; HFRT, hypofractionated radiotherapy; LD-CYC, low-dose cyclophosphamide; PD-L1, programmed death-ligand 1; CTLA-4, cytotoxic T-lymphocyte-associated protein 4; CR, complete response; ORR, objective response rate; mPFS, median progression-free survival; OS, overall survival; EFS, event-free survival; HR, hazard ratio; TMB, tumor mutational burden; irAE, immune-related adverse event; Gr, grade; TRAE, treatment-related adverse event; mDOR, median duration of response; CRL, complete response letter; FDA, US, food and drug administration; pDC, plasmacytoid dendritic cell; CTL, cytotoxic T lymphocyte; LN, lymph node; OXPHOS, oxidative phosphorylation; HLA, human leukocyte antigen; DC, dendritic cell; APC, antigen-presenting cell; CRT, calreticulin; ATP, adenosine triphosphate; HMGB1, high mobility group box 1; ICD, immunogenic cell death; SDT, sonodynamic therapy; ROS, reactive oxygen species; CDT, chemodynamic therapy; BSO, L-buthionine sulfoximine; CuO2, copper oxide; GEL, thermosensitive hydrogel; ERp57, endoplasmic reticulum protein 57; eIF2α, eukaryotic translation initiation factor 2 alpha; TRAEs, treatment-related adverse events; DCR, disease control rate; mfg, manufacturin.

### 3.4 Critical appraisal: evidence hierarchy, treatment sequencing, and tumor heterogeneity

#### 3.4.1 Evidence hierarchy

Ziogas et al. (2023) systematically delineated the multidimensional challenges in overcoming PD-1/PD-L1 inhibitor resistance in melanoma, emphasizing that while a breadth of reversal strategies demonstrates preclinical or early clinical promise, their clinical maturity remains highly variable. CTLA-4 plus PD-1 blockade, BRAF/MEK inhibitors plus PD-1 inhibitors, and TIL therapy (lifileucel) are supported by phase III or registrational trials, representing established or near-established clinical approaches (Larkin et al., 2015; Ribas et al., 2019; Sarnaik et al., 2021). For talimogene laherparepvec plus pembrolizumab, phase 1b proof-of-concept data are available (Ribas et al., 2017), with definitive phase III evidence coming from the MASTERKEY-265 trial (Chesney et al., 2023). In contrast, STING agonists, copper-targeted therapies, PARP14 inhibitors, SDH inhibitors, and METTL3 modulators remain predominantly preclinical, with limited translational validation to date (Nakamura et al., 2021; Wong et al., 2023; Chrzan and Hartman, 2025; Meraz-Torres et al., 2024; Liang et al., 2025; Wu et al., 2024). Overstating the translational readiness of early-phase findings risks misleading clinical decision-making—a concern explicitly raised by reviewers evaluating this field. Notably, RP1 (vusolimogene mwaenigenia) plus nivolumab demonstrated a 32.9% ORR in PD-1-refractory melanoma within the single-arm phase 2 IGNYTE trial (Wong et al., 2025). However, the US FDA issued a Complete Response Letter in July 2025, citing the absence of randomized controlled data and insufficient evidence of sustained clinical benefit to support full regulatory approval. This disconnect between encouraging early-phase efficacy and the rigor required for registrational endpoints underscores a broader translational bottleneck: single-arm phase II results, while hypothesis-generating, often fail to meet the evidentiary bar for routine clinical adoption without subsequent randomized validation.

#### 3.4.2 Treatment sequencing

In BRAF-mutant melanoma, the optimal sequence of targeted therapy versus immunotherapy remains actively debated. Upfront triplet combination (BRAF/MEK inhibitors plus PD-1 blockade) achieves deep, durable responses but incurs overlapping toxicity profiles that limit tolerability in unfit patients (Ribas et al., 2019). Sequential approaches—either targeted therapy followed by immunotherapy, or vice versa—may mitigate cumulative adverse events but carry the risk of clonal selection of resistant subpopulations during treatment gaps. For PD-1-refractory disease, TIL therapy offers a validated salvage option with a 31.4% ORR in registrational cohorts (Sarnaik et al., 2021), while RP1 plus nivolumab provides a less resource-intensive alternative with comparable efficacy, albeit with pending regulatory clarity as noted above (Wong et al., 2025). The choice between these modalities hinges on real-time tumor burden kinetics, patient fitness for lymphodepleting preconditioning (required for TIL therapy), and institutional access to cell manufacturing infrastructure.

#### 3.4.3 Tumor heterogeneity

Resistance to PD-1/PD-L1 inhibitors is fundamentally shaped by clonal evolution and spatial heterogeneity, a reality often underappreciated in preclinical modeling (Morad et al., 2021). Single-cell and spatial transcriptomic studies reveal that distinct resistance sub-clones coexist within a single tumor: some clones lose antigen presentation machinery (e.g., B2M mutations), while others remodel the TME to promote immunosuppression (e.g., MDSC infiltration, TGF-β secretion). This intratumoral heterogeneity explains why single-agent therapies achieve only modest response rates in unselected populations and underscores the necessity of rationally designed combinations that target multiple resistance layers simultaneously—for example, pairing antigen presentation-restoring agents with TME-depleting therapies to address coexisting resistance clones (Morad et al., 2021).

#### 3.4.4 Toxicity management

Combination regimens invariably amplify on-target and off-target toxicities relative to monotherapy. CTLA-4 plus PD-1 blockade incurs grade 3–4 treatment-related adverse events in 59% of patients, requiring high-intensity immunosuppressive intervention in a subset (Larkin et al., 2015). Adoptive cell therapies such as TIL are associated with cytokine release syndrome and prolonged cytopenias linked to lymphodepleting preconditioning (Sarnaik et al., 2021). Even preclinical approaches such as systemic copper chelation carry risks of disrupting systemic copper homeostasis, with poorly defined therapeutic windows (Chrzan and Hartman, 2025). Biomarker-driven patient selection (e.g., excluding patients with autoimmune comorbidities from intensive combination regimens) and proactive monitoring/intervention for immune-related adverse events remain central to the safe translation of reversal strategies into routine clinical practice. Notably, several unresolved contradictory observations persist across current translational research, which deserve balanced critical discussion. First, the irreversible resistance induced by B2M loss is not absolute across all patient cohorts: while bulk sequencing data confirm complete MHC-I loss abolishes CD8^+^ T cell recognition in most lesions, small retrospective cohorts reveal partial tumor regression after NK-cell-based combinational therapy in patients with heterozygous B2M inactivation, indicating residual antigen presentation machinery can be partially rescued, a discrepancy rarely emphasized in previous publications (Sade-Feldman et al., 2017; Torrejon et al., 2023). Second, gut microbiota intervention via FMT yields highly variable clinical response across independent cohorts; inconsistent donor screening criteria, baseline intestinal immune status of recipients, and prior antibiotic exposure collectively confound therapeutic efficacy, creating contradictory trial outcomes that limit standardized clinical adoption (Simiczyjew et al., 2020; Davar et al., 2021). Third, HDAC inhibitors exert dual modulatory effects on tumor immune phenotype: aside from upregulating MHC-II expression to restore anti-tumor immunity, these agents simultaneously boost tumoral PD-L1 abundance, generating context-dependent therapeutic trade-offs that complicate optimal dosing and treatment sequencing (James et al., 2023; Woods et al., 2015). Collectively, these conflicting findings highlight that single-mechanism therapeutic reasoning cannot fully explain clinical response heterogeneity.

## 4 Biomarkers and patient stratification

Exploration and application of biomarkers are the key to precise treatment of melanoma with PD-1/PD-L1 inhibitors, which can provide an important basis for patient stratification, efficacy prediction and early warning of resistance. According to different clinical application stages, existing biomarkers can be divided into three categories, corresponding to different resistance mechanisms and clinical scenarios.

### 4.1 Predictive biomarkers for anti-PD-1-directed immunotherapy in melanoma

This section focuses on predictive biomarkers that forecast therapeutic responses to anti-PD-1 immunotherapy in melanoma, which are mechanistically and clinically distinct from tumor diagnostic biomarkers. PD-L1 immunohistochemistry is routinely applied in clinical practice for patient stratification. In contrast, TMB and TIL density remain investigational biomarkers; they are not standard-of-care clinical assays for melanoma management, nor are they validated routine biomarkers for melanoma.

PD-L1 expression is the most widely adopted predictive biomarker in clinical practice. Tumors with elevated PD-L1 expression on malignant or immune cells exhibit superior responsiveness to PD-1/PD-L1 blockade, consistent with the canonical PD-L1-mediated tumor immune evasion mechanism (Richmond et al., 2019). Beyond PD-L1 status, TMB serves as a core tissue biomarker reflecting tumor neoantigen-generating potential. High-TMB melanomas exhibit enhanced immunogenicity and improved anti-PD-1 therapeutic efficacy, representing a key indicator of intrinsic tumor immunogenicity (Kalaora et al., 2020). Similarly, CD8^+^ T-cell infiltration density and the CD8^+^/Treg ratio characterize the immune landscape of the melanoma TME. Robust CD8^+^ T-cell infiltration and an elevated CD8^+^/Treg ratio indicate an immune-active TMEs and are associated with enhanced antitumor T-cell activation, including oncolytic-virus-driven remodeling of the TME (Chen et al., 2021).

### 4.2 Biomarkers in clinical validation stage

Several biomarkers have been verified by many clinical cohorts, have the potential to enter clinical application, and can be used for precise typing and efficacy prediction.

B2M mutation status is a key predictive biomarker for antigen presentation-deficient resistance. In metastatic melanoma lesions with acquired resistance to PD-1 inhibitors, the incidence of B2M mutation is significantly higher than that in primary lesions; loss of MHC-I molecule surface expression caused by B2M mutation is an important mechanism of PD-1 inhibitor resistance (Richmond et al., 2019). Sequential treatment failures involving BRAF/MEK inhibitors followed by immune checkpoint blockade are also frequently mediated by emergent MAP2K2 and B2M mutations (Richmond et al., 2019). Immunoproteasome catalytic subunits (PSMB8/PSMB9) are upregulated in responsive tumors, enhancing TIL reactivity and correlating with improved checkpoint blockade outcomes (Kalaora et al., 2020). TAM-targeted nanocomplexes that reprogram the immunosuppressive microenvironment represent a delivery/platform strategy (Han et al., 2021). However, the use of such a strategy in melanoma requires melanoma-specific validation. Melanoma-specific MHC-II expression constitutes a tumor-autonomous phenotype predictive of anti-PD-1/PD-L1 response, independent of conventional immune infiltrate metrics (Johnson et al., 2016). M2-like TAM polarization enriches immunosuppressive signaling in the TME across multiple cancers (Boutilier and Elsawa, 2021). In melanoma, TAM phenotypic markers are plausible resistance biomarkers that require further investigation in melanoma tumor models.

Serial measurement of circulating tumor DNA (ctDNA) permits real-time surveillance of therapeutic response across solid tumors (Bratman et al., 2020). In melanoma, ctDNA could also be informative in predicting treatment response, which necessitates further investigations. IFN-γ-related transcriptional signatures predict checkpoint-blockade outcomes across various cancer types, including melanoma (Liu et al., 2023). Baseline or on-treatment serum IFN-γ levels further refine this predictive capacity (Liu et al., 2023). FDG-PET metabolic response can accurately predict pathological response and recurrence-free survival of neoadjuvant immunotherapy. Among patients showing >70% reduction in SUVmax, 96% achieved a major pathological response (MPR), and the recurrence rate in this subgroup was only 4.4% (Zhou et al., 2025). Understanding the distinct mechanisms of action and limitations of anti-PD-1 versus anti-CTLA-4 therapies informs the interpretation of these FDG-PET-derived metabolic imaging biomarkers (e.g., SUVmax). Distinct immune-activation patterns and pseudo-progression profiles under anti-PD-1 and anti-CTLA-4 treatment may alter PET metabolic signals; awareness of these mechanistic differences helps to avoid mis-classifying imaging changes as true tumor progression (Seidel et al., 2018). Intestinal microbiota composition can be used as a core biomarker for systemic resistance prediction in melanoma. Enrichment of Ruminococcaceae, Bifidobacteriaceae and Lachnospiraceae in the intestine indicates better treatment response to anti-PD-1 therapy (McCulloch et al., 2022). Meta-analyses confirm that tumor-intrinsic and T-cell-extrinsic mechanisms collectively dictate sensitivity to checkpoint inhibition (Litchfield et al., 2021). Intra- and intertumoral heterogeneity further complicates biomarker interpretation, as spatially distinct clones may harbor divergent resistance alterations (Grzywa et al., 2017).

Four soluble biomarkers (soluble PD-L1, sCTLA-4, sCD73, and sCD74) independently predict progression-free and overall survival in metastatic melanoma patients; a composite risk score derived from sCTLA-4 and sCD74 outperforms conventional serological metrics (Azzariti et al., 2025). For uveal melanoma—a TMB-low subtype with historically poor immunotherapy response—the 2394-gene Uveal Melanoma Immunoscore (UMIS) identifies occult T cell-inflammatory signals predictive of response to autologous TIL therapy, broadening biomarker utility to immunologically cold malignancies (Leonard-Murali et al., 2024).

### 4.3 Biomarkers in preclinical exploration stage

Various biomarkers are being investigated in the preclinical phases. These biomarkers are being mainly explored in animal studies and in small-scale human studies. They require further clinical validation in large-scale studies to direct future stratification systems.

STING promoter methylation status can predict the efficacy of oncolytic virus combined with immunotherapy. Non-injected lesions in STING-low patients have 100% regression, corresponding to the resistance mechanism of STING pathway silencing (Fang et al., 2025). Low expression of immunoproteasome catalytic subunits (PSMB8/PSMB9) indicates poor response to ICIs, while overexpression can enhance the reactivity of TILs, corresponding to the resistance mechanism of antigen processing and presentation (Kalaora et al., 2020). The CD11b^+^PD-L1^+^ Breg-derived four-gene signature (ITGAM, CD274, TNFRSF13B, AHNAK) is associated with poor overall survival in melanoma patients and can predict therapeutic response to RAS-pathway-targeted inhibitors combined with CD40 agonism (Yan et al., 2026). Serum biomarkers such as serum B2M and baseline serum IFN-γ levels are non-invasive auxiliary evaluation indicators, which can indirectly predict treatment benefit and disease progression (Liu et al., 2023).

Recent spatial multi-omics analyses have delineated nine pan-cancer-conserved spatial ecotypes (SEs), recurrent multicellular tissue niches defined by distinct spatial localization and cellular composition, by integrating 132 primary-tumor spatial transcriptomes and 144 single-cell RNA-seq datasets (Zhang et al., 2026). Localized primarily at tumor-stromal boundaries, the SE7 spatial ecotype accumulates abundant effector immune cell subsets, whereas SE8 occupies intratumor core regions and similarly contains large pools of anti-tumor-directed immune cells. Analyses covering 1249 pre-treatment tumor samples demonstrated that higher SE7 and SE8 signature scores independently forecast sustained clinical gains under anti-PD-1 therapy (PFS HR < 0.38, P < 0.001), outperforming established biomarkers such as TMB, PD-L1 expression and the Tumor Immune Dysfunction and Exclusion (TIDE) model. In sharp contrast, SE4 constitutes a suppressive tissue niche dominated by myofibroblasts and hypoxia-related endothelial cells together with immunosuppressive myeloid infiltrates; elevated SE4 signatures correlate with robust primary immunotherapy refractoriness (PFS HR = 2.92) (Zhang et al., 2026). The Liquid EcoTyper computational tool allows inference of these spatial ecotypes from pre-treatment plasma cell-free DNA, removing the dependence on tumor tissue biopsy (Zhang et al., 2026). Despite robust predictive performance within internal study cohorts, these spatial multi-omics and machine-learning-based approaches still face clinical-translation barriers: multiplex immunofluorescence and spatial-transcriptomic platforms are not routinely available in community-based pathology laboratories, and prediction algorithms trained on single-center datasets lack broad external validation across multi-ethnic melanoma cohorts (Zhang et al., 2026; Pybus et al., 2026). Complementing these transcriptomic and spatial profiling findings, post-translational modification-based biomarkers such as ubiquitin-specific peptidase 5 (USP5)-mediated PD-L1 stabilization further refine risk stratification when integrated into a multi-gene model: a 48-gene post-translational modification risk model (PTMRM) incorporating USP5 outperforms conventional biomarkers in predicting immunotherapy outcomes across TCGA-SKCM and three independent GEO cohorts (Xie et al., 2025).

### 4.4 Development and integration of multi-biomarker approach

The predictive efficacy of a single biomarker is limited. In clinical practice, melanoma resistance is often driven by multiple mechanisms, so multi-biomarker integration model is the core direction of precise stratification in the future. Existing studies have shown that multi-dimensional assessment combining TMB, PD-L1 expression and CD8^+^ T cell infiltration can predict immunotherapy response more accurately than a single indicator (Richmond et al., 2019; Kalaora et al., 2020). Intra- and intertumoral heterogeneity further complicates biomarker interpretation, necessitating integrated approaches (Grzywa et al., 2017). In the future, multi-omics integration models based on intrinsic tumor characteristics, microenvironment state and systemic immune level are expected to further improve prediction accuracy and provide individualized treatment plans for patients (Zhang et al., 2026; Pybus et al., 2026). Machine learning algorithms trained on multiplexed imaging and multi-omics datasets will be pivotal in decoding these complex interactions (Pybus et al., 2026).

Clinical decision-making must also incorporate real-world complexities beyond biomarker status alone. In patients with melanoma brain metastases, 84.3% discontinue combination immunotherapy within 24 months for reasons unrelated to disease progression. Among patients achieving a complete response who stop treatment because of immune-related toxicity, the 4-year overall survival reaches 89.6%. Notably, immune-related adverse events are independently linked to prolonged survival in the non-anti-TNFα subgroup (HR 0.18) (Mandalà et al., 2026). The evolution of melanoma therapy from targeted agents to immunotherapy to rational combinations highlights the need for biomarkers tailored to novel regimens, including mRNA vaccines which exhibit favorable safety profiles in early-phase trials (Flaherty et al., 2025). Overcoming resistance demands a shift from static, single-biopsy snapshots to dynamic, multi-omics surveillance frameworks that adapt to clonal evolution and spatial remodeling throughout the treatment course (Mandal et al., 2025). Reprogramming the TME to convert “cold” tumors to “hot” via targets such as LAG-3, TIM-3, TAM MARCO, and ERRα is a broadly articulated strategic direction in immune-oncology (Yuan et al., 2025). Such a strategy can be further investigated in melanoma. Concurrent stereotactic radiotherapy with dual immunotherapy modulates the TME, yet steroid use in this setting independently worsens survival (HR 1.56), underscoring the interplay between systemic management and biomarker interpretation (Mandalà et al., 2025). Clinical development of novel immuno-oncology therapeutics, including TIL, TCR-T, and mRNA vaccines, continues to expand the armamentarium for biomarker-selected populations (Wang et al., 2025). Neoadjuvant treatment sequencing represents another frontier for biomarker application: the NADINA trial demonstrated that neoadjuvant nivolumab plus ipilimumab followed by adjuvant therapy achieved a superior event-free survival of 83.7% compared to adjuvant nivolumab alone (57.2%), establishing a new benchmark for biomarker-driven surgical sequencing (Blank et al., 2024).

Table 4 summarizes the core indicators, predictive value and application scenarios of the above-described biomarkers.

**TABLE 4:** Hierarchical biomarker system for stratification of patients and resistance prediction in melanoma.

Biomarker	Sample type	Resistance mechanism	Validation status	Applicable melanoma subtypes	Clinical applicability	Standardization/limitations	References
PD-L1 expression	Tumor/immune cells (IHC)	PD-L1-mediated T cell inhibition	Routine clinical	Cutaneous all subtypes	First-line stratification; guides monotherapy vs. combo	Assay/platform variability; dynamic changes; cutoff debate	Richmond et al. (2019)
TMB (high)	Tumor tissue (NGS)	Tumor immunogenicity	Routine clinical	Cutaneous; limited utility for uveal/acral	Predicts response to PD-1 inhibitors; FDA-approved companion Dx	TMB-low tumors can respond; assay standardization; tumor-type specific	Kalaora et al. (2020)
B2M mutation/MHC-I loss	Tumor tissue (NGS/IHC)	Antigen presentation defect	Clinical validation	Cutaneous melanoma (rare in uveal)	Predicts acquired resistance to PD-1 inhibitors; repeat biopsy guidance	Clonal heterogeneity; not all resistant tumors lose MHC-I	Richmond et al. (2019)
PSMB8/PSMB9 (immunoproteasome)	Tumor tissue (RNA/protein)	Antigen processing and presentation	Clinical validation	Cutaneous; partial value for acral	Complements TMB; predicts checkpoint response	Assay not routine; prospective validation needed	Kalaora et al. (2020)
MHC-II (tumor-specific)	Tumor tissue (RNA/IHC)	Tumor-autonomous immune evasion	Clinical validation	Cutaneous only	Independent of infiltrate metrics; predicts anti-PD-1 response	Confirmatory cohorts needed	Johnson et al. (2016)
IFN-γ mRNA signature	Tumor tissue (RNA)	IFN-γ pathway/T cell inflammation	Clinical validation	Cutaneous; weak for uveal	Predicts PD-1 inhibitor response; serum IFN-γ augments	Gene-panel definition variable; validation cohorts needed	Liu et al. (2023)
FDG-PET (SUVmax reduction)	Imaging (PET/CT)	Treatment response (neoadjuvant)	Clinical validation	Pan-melanoma universal	Predicts MPR and RFS; 96% MPR if ΔSUVmax>70%	Standardized acquisition protocols; cost	Zhou et al. (2025)
Gut microbiota composition	Stool (16S/metagenomics)	Systemic immune modulation	Clinical validation	Pan-melanoma universal	Predicts response; FMT as rescue therapy	Donor selection heterogeneity; long-term safety data lacking	McCulloch et al. (2022)
Four-marker liquid biopsy (sPD-L1, sCTLA-4, sCD73, sCD74)	Serum (ELISA/multiplex)	Systemic immune status	Clinical validation	Pan-melanoma universal	Risk score for PFS/OS; sCTLA-4+sCD74-derived score	Pre-analytical variables; validation in prospective trial	Azzariti et al. (2025)
UMIS (uveal melanoma)	Tumor tissue (RNA)	“Cold” TME in uveal melanoma	Clinical validation	Uveal melanoma exclusive	Identifies T cell-inflamed subset; guides TIL therapy	Uveal-melanoma specific; rare subtype	Leonard-Murali et al. (2024)
Tumor heterogeneity indices	Tumor tissue (NGS/RNA)	Clonal evolution and divergence	Clinical validation	Pan-melanoma universal	Highlights need for combo; explains single-agent failure	Single-biopsy snapshot; spatial sampling bias	Grzywa et al. (2017)
STING promoter methylation	Tumor tissue (methylation array)	STING pathway silencing	Preclinical	Cutaneous only	Predicts oncolytic virus + ICI efficacy	Assay not clinical-grade	Fang et al. (2025)
CD11b+PD-L1+ Breg signature	Tumor/blood (RNA)	Breg-mediated suppression	Preclinical	NRAS-mutant cutaneous only	Predicts RAS inhibitor + CD40 agonist efficacy	Discovery cohort only; NRAS-specific	Yan et al. (2026)
Serum B2M/IFN-γ	Serum (ELISA)	Systemic immune state	Preclinical	Pan-melanoma universal	Non-invasive auxiliary prognosis	Indirect; needs prospective validation	Liu et al. (2023)
Spatial ecotypes (SE7/SE8/SE4)	Tumor FFPE/plasma cfDNA	Spatial TME architecture	Preclinical → early clinical	Pan-melanoma universal	SE7/SE8 predict benefit (PFS HR < 0.38); SE4 predicts resistance (PFS HR = 2.92)	Novel assay; prospective validation pending	Zhang et al. (2026)
RCN8 cellular neighborhood	Tumor FFPE (16-plex IF)	Spatial immune topology	Preclinical	Cutaneous primary lesions	AUC 0.76 for response prediction	16-plex mIF high cost; computational pipeline complex	Pybus et al. (2026)
USP5/PTMRM 48-gene model	Tumor tissue (RNA/multiplex)	Post-translational PD-L1 stabilization	Preclinical	Cutaneous melanoma	Outperforms conventional biomarkers in multi-cohort validation	Assay development; prospective trials needed	Xie et al. (2025)
Multi-biomarker integration (TMB + PD-L1+CD8^+^)	Tissue + blood + imaging	Multi-dimensional resistance	Clinical validation	Pan-melanoma universal	Superior to single markers; foundation for precision stratification	Algorithm standardization; data integration infrastructure	Richmond et al. (2019), Kalaora et al. (2020)
NADINA trial neoadjuvant regimen	Clinical trial (FFPE/plasma)	Treatment timing optimization	Clinical validation (Ph III)	Resectable stage III cutaneous	EFS 83.7% vs. single adjuvant immunotherapy	Restricted to resectable disease; no standalone molecular biomarker identified	Blank et al. (2024)

Evidence grading standards are consistent with Table 1. Abbreviations: PD-L1, programmed death-ligand 1; IHC, immunohistochemistry; TMB, tumor mutational burden; NGS, next-generation sequencing; B2M, β2-microglobulin; MHC-I, major histocompatibility complex class I; MHC-II, major histocompatibility complex class II; PSMB8/9, immunoproteasome subunit beta type 8/9; ISG, interferon-stimulated gene; IFN-γ, interferon-gamma; ctDNA, circulating tumor DNA; ddPCR, droplet digital polymerase chain reaction; FDG-PET, fluorodeoxyglucose positron emission tomography; SUVmax, maximum standardized uptake value; MPR, major pathological response; RFS, recurrence-free survival; TAM, tumor-associated macrophage; TME, tumor microenvironment; sPD-L1, soluble programmed death-ligand 1; sCTLA-4, soluble cytotoxic T-lymphocyte-associated protein 4; sCD74, soluble cluster of differentiation 74; sCD73, soluble cluster of differentiation 73; ELISA, enzyme-linked immunosorbent assay; PFS, progression-free survival; OS, overall survival; UMIS, uveal melanoma immune signature; FFPE, formalin-fixed paraffin-embedded; cfDNA, cell-free DNA; SE, spatial ecotype; HR, hazard ratio; AUC, area under the curve; mIF, multiplex immunofluorescence; USP5, ubiquitin-specific peptidase 5; PTMRM, post-translational modification risk model; TCGA, the cancer genome atlas; GEO, gene expression omnibus; EFS, event-free survival; NRAS, neuroblastoma RAS viral oncogene homolog; BRAF, B-Raf proto-oncogene; TIL, tumor-infiltrating lymphocyte; FMT, fecal microbiota transplantation; ICI, immune checkpoint inhibitor.

## 5 Discussion and conclusion

### 5.1 Discussion

Resistance to PD-1/PD-L1 inhibitors in melanoma represents a dynamically evolving, multi-dimensional process in which tumor-intrinsic defects, immunosuppressive microenvironment remodeling, metabolic rewiring, and systemic modulators engage in bidirectional crosstalk. Reflecting this complexity, contemporary research has shifted from reductionist focus on single molecules toward integrative analyses of multifactorial interactive regulatory networks, rendering single-target interventions insufficient to durably reverse resistance in clinical practice. Multiple lines of evidence, including meta-analyses of trial cohorts, confirm that rationally designed combination strategies—simultaneously targeting intrinsic oncogenic drivers and adaptive immune evasion—yield superior efficacy over single-agent approaches (Litchfield et al., 2021; Yuan et al., 2025).

The development of current resistance reversal strategies has made phased progress, but still faces three core challenges. First, tumor heterogeneity limits the efficacy of single regimens. Melanoma itself has the characteristics of high TMB and high clonal heterogeneity. Different lesions of the same patient and different cell subsets in the same lesion may have different resistance mechanisms, making it difficult for a single treatment to cover all resistant clones (Grzywa et al., 2017); melanoma with different mutation subtypes such as BRAF and NRAS also have significant differences in dominant resistance mechanisms, which further increases the difficulty of treatment (Yan et al., 2026; Yuan et al., 2025). Second, toxic superposition of combination therapy limits clinical application. Although dual immune combination, targeted-immune triple therapy and other regimens improve efficacy, the incidence of grade 3–4 adverse reactions is significantly increased, and some elderly patients or patients with underlying diseases cannot tolerate the complete treatment cycle, ultimately affecting long-term benefit (Morad et al., 2021; Yuan et al., 2025). Real-world clinical observations from melanoma brain metastasis cohorts show that 84.3% of patients discontinue combination immunotherapy within 24 months for reasons other than disease progression (Mandalà et al., 2026). Among patients achieving complete response who stop treatment because of treatment-related toxicity, the 4-year overall survival reaches 89.6% (Mandalà et al., 2026). Of note, concomitant steroid use independently confers poorer survival outcomes (HR 1.56) in this patient population (Mandalà et al., 2025). Third, the universality and accuracy of biomarkers are still insufficient. At present, no single biomarker can accurately predict the treatment response of all patients. Most novel biomarkers are still in the stage of small sample exploration, lacking large sample prospective validation; and most existing biomarkers focus on local tumors, with limited ability to evaluate systemic immune state, making it difficult to achieve full-dimensional precise stratification (Morad et al., 2021; Mandal et al., 2025; Yuan et al., 2025). However, emerging technologies are beginning to address these gaps: spatial ecotypes (SE7/SE8 predicting benefit, SE4 predicting resistance) and spatially aware machine learning models (AUC 0.76) enable non-invasive, system-level immune profiling (Zhang et al., 2026; Pybus et al., 2026). As described previously, the NADINA trial demonstrated that the combined neoadjuvant nivolumab and ipilimumab treatment followed by adjuvant therapy was superior to adjuvant nivolumab alone (Blank et al., 2024).

In addition, problems such as neutralizing antibody clearance and difficulty in penetrating matrix barriers faced by oncolytic virus therapy (Ribas et al., 2017), as well as high preparation cost and complex individualized procedures faced by cell therapy (Sarnaik et al., 2021), further limit the clinical popularization of related strategies (Yuan et al., 2025). The translational pipeline must also bridge preclinical discoveries with clinical realities (Mandal et al., 2025; Yuan et al., 2025)—while USP5 inhibitors and spatial ecotype-based models show promise in murine and computational models (Zhang et al., 2026; Xie et al., 2025), their clinical translation necessitates rigorous pharmacokinetic profiling and biomarker-selected trial designs.

#### 5.1.1 Future directions

Future research needs to further focus on the interwoven regulatory network of multiple mechanisms, develop more precise combination treatment regimens, and improve the multi-dimensional biomarker system to enhance personalized immunotherapy (Mandal et al., 2025; Yuan et al., 2025). Three core unsolved scientific bottlenecks continue to hinder the full clinical realization of melanoma immunotherapy precision: (1) the dynamic crosstalk between intratumoral clonal evolution and systemic intestinal immunity remains poorly quantified through longitudinal multi-omics monitoring; (2) subtype-specific multidimensional resistance networks for acral, mucosal and uveal melanoma lack large cohort prospective validation; (3) uniform standardized detection protocols for spatial transcriptomic and liquid biopsy biomarkers have not been established across clinical laboratories. Resolving these three gaps will be a prerequisite for shifting from empirical combination regimens to mechanism-guided individualized treatment (Mandal et al., 2025; Yuan et al., 2025).

### 5.2 Conclusion

Multiple combinatorial therapeutic modalities have been developed to tackle immunotherapy resistance in melanoma. Biomarkers constitute the core bridge connecting mechanistic understanding of resistance and real-world clinical decision-making. A multi-dimensional biomarker framework is gradually taking shape, spanning routinely used clinical indicators, markers undergoing clinical validation, and exploratory signatures including spatial ecotypes, post-translational modification read-outs and artificial-intelligence-driven predictive tools. Multi-biomarker integrated approaches represent an essential path toward refined patient stratification for melanoma immunotherapy.

Overcoming immunotherapy resistance requires improved comprehension of multidimensional mechanistic crosstalk as well as prospective clinical validation of combinatorial treatment strategies. Advancement toward mechanism-guided individualized melanoma management will rely on multimodal analytical resources, including spatial profiling, liquid-biopsy assays and machine-learning-assisted analytical workflows, to improve long-term patient outcomes.

## Glossary

ACT: adoptive cell therapy
B2M: β2-microglobulin
Bregs: regulatory B cells
cGAS: cyclic GMP-AMP synthase
CAR-iNKT: chimeric antigen receptor invariant natural killer T cell
CCR5: C-C motif chemokine receptor 5
CPT1A: carnitine palmitoyltransferase 1A
CRL: Complete Response Letter
ctDNA: circulating tumor DNA
CTLA-4: cytotoxic T-lymphocyte-associated protein 4
DAMPs: damage-associated molecular patterns
DDTC: diethyldithiocarbamate
DC: dendritic cell
DMM: dimethyl malonate
DSF: disulfiram
EZH2: enhancer of zeste homolog 2
FDG-PET: fluorodeoxyglucose positron emission tomography
FMT: fecal microbiota transplantation
G-CSF: granulocyte colony-stimulating factor
GM-CSF: granulocyte-macrophage colony-stimulating factor
HDAC: histone deacetylase
HLA-I: human leukocyte antigen class I
ICD: immunogenic cell death
ICI: immune checkpoint inhibitor
IFN-γ: interferon-gamma
IL: interleukin
iNKT: invariant natural killer T cell
IRF1: interferon regulatory factor 1
LAG-3: lymphocyte activation gene 3
MDSCs: myeloid-derived suppressor cells
mDOR: median duration of response
mIF: multiplex immunofluorescence
MHC: major histocompatibility complex
MITF: microphthalmia-associated transcription factor
NK cell: natural killer cell
ORR: objective response rate
PD-1: programmed cell death protein 1
PD-L1: programmed death-ligand 1
PGE2: prostaglandin E2
PRC2: polycomb repressor complex 2
PARP14: poly(ADP-ribose) polymerase 14
PSMB: immunoproteasome subunit beta type
PTMRM: post-translational modification risk model
SE: spatial ecotypes
STING: stimulator of interferon genes
TAP: transporter associated with antigen processing
TAMs: tumor-associated macrophages
TCF4: transcription factor 4
TCR-T: T-cell receptor engineered T cell
TIDE: Tumor Immune Dysfunction and Exclusion
TIL: tumor-infiltrating lymphocyte
TMB: tumor mutational burden
Treg: regulatory T cell
T-VEC: talimogene laherparepvec
UMIS: Uveal Melanoma Immunoscore
USP5: ubiquitin-specific peptidase 5
